# The tumor-suppressive effects of alpha-ketoglutarate-dependent dioxygenase FTO via N6-methyladenosine RNA methylation on bladder cancer patients

**DOI:** 10.1080/21655979.2021.1964893

**Published:** 2021-09-09

**Authors:** Wenfa Yi, Yong Yu, Yafei Li, Juan Yang, Shuying Gao, Lingfen Xu

**Affiliations:** a Department Of Urology, Qinghai Provincial People’s Hospital, Xining, Qinghai Province, China; b Department Of Oncology, Qinghai Provincial People’s Hospital, Xining, Qinghai Province, China; c Department Of Endoscopy Room, Qinghai Provincial People’s Hospital, Xining, Qinghai Province, China; d Department Of General Practice, Qinghai Provincial People’s Hospital, Xining, Qinghai Province, China

**Keywords:** FTO, bladder cancer, m6A rna methylation, sfpq

## Abstract

N6-methyladenosine (m6A) methylation participates in the progression of bladder cancer (BCa). Nevertheless, the regulatory mechanism of alpha-ketoglutarate-dependent dioxygenase FTO influencing the BCa progression has still remained elusive. In this study, to investigate the tumor-suppressive effects of FTO via m6A RNA methylation on BCa patients, a total of 15 cancer tissues and adjacent normal tissues (ANTs) were collected from BCa patients who received tumor resection in our hospital from September 2015 to December 2019. We found that the FTO expression was significantly reduced in cancer tissues compared with that in ANTs, which indicated a lower malignant potential and a higher overall survival rate. It was revealed that overexpression of FTO in two human urinary BCa cell lines (HT-1197 and HT-1376) significantly decreased the cell proliferation and invasion abilities compared with the negative controls, whereas the cell apoptosis was markedly enhanced. In addition, we noted that the changes in m6A methylation level mainly appeared at 5ʹ untranslated region (5ʹ UTR) of MALAT1 and NOTCH1 transcripts, and at 3ʹ UTR of CSNK2A2 and ITGA6 transcripts, responding to the overexpression of FTO. Mechanistically, we found that the splicing factor, proline- and glutamine-rich (SFPQ) could influence the FTO-mediated m6A RNA demethylation, eventually affecting the gene expression. This study provided a new insight into the relationship between the FTO expression and the m6A RNA methylation, assisting scholars to better understand the pathogenesis of BCa.

## Introduction

Bladder cancer (BCa), one of the most common malignancy of the urinary tract, is characterized by high rates of incidence, prevalence, recurrence, and mortality [1]. Genetic, epigenetic, and environmental factors are involved in the etiology of BCa [2]. Despite significant advances achieved in the treatment of diverse types of cancer by surgery and chemotherapy, no effective therapeutic approach for BCa has been presented yet, which may be related to the late diagnosis and high incidence of chemoresistance [3]. In recent decades, a large number of scholars have concentrated on developing new therapeutic strategies to overcome challenges originating from chemoresistance [4]. In addition to the necessity of development of novel diagnostic approaches, the molecular mechanisms underlying BCa need to be deeply studied to explore further effective therapeutic targets for BCa.

Recent findings have revealed that N6-methyladenosine (m6A) RNA methylation is prevalently and abundantly enriched in eukaryotic cells [5]. A number of scholars pointed out that m6A RNA methylation has been found to be involved in multiple cellular processes, such as RNA maturation, alternative splicing, and protein synthesis [6,7]. The growing evidence suggested that abnormally regulation of m6A may profoundly contribute to the carcinogenesis [8,9]. A series of enzymes termed as m6A ‘writers’, ‘erasers’, and ‘readers’ based on the different functions have been proposed to participate in m6A RNA methylation modification. The fat mass and obesity-associated protein (FTO), as the first RNA demethylase, has made a great evolution in RNA methylation research, and it was revealed that m6A is a reversible and dynamic RNA modification that may influence biological regulation analogous to the well-studied reversible DNA and histone modifications [10]. A recent study demonstrated that FTO is beneficial to cisplatin-induced cytotoxic effects on patients with BCa [11]. FTO may contribute to demethylation of RNAs in nucleus, because of its localization and enrichment in nuclear speckles, although RNAs can be demethylated at N6-methyladenosine sites by FTO [12]. Approximately, a quarter of transcripts contain m6A sites at 5ʹ untranslated region (5ʹ UTR), 3ʹ UTR, exons, and stop codons with the consensus motifs of ‘GAC’ and ‘AAC’[13]. 5ʹ UTR m6A residues enable a cap-independent model of the translation initiation via bypassing the m7G cap requirement [14]. Mechanistically, R-2HG increases m6A RNA methylation through suppression of FTO activity, destabilizing MYC/CEBPA transcripts in leukemia cells [15]. The enrichment of m6A in 3ʹ terminal of mature polyadenylated mRNAs is associated with an alternative polyadenylation [5]. On the contrary, the intracellular level of S-adenosylmethionine (SAM) in mammalian cells is partly regulated by SAM-responsive RNA degradation of MAT2A mRNA [16]. However, synergistic effects of m6A RNA methylation at different sites on the gene expression have still remained elusive. The coordination of enzymes for m6A RNA methylation with other transcription or translation factors in multiple human diseases is extremely complicated.

In the present study, we assessed the tumor-inhibitory effects of FTO via m6A RNA methylation on BCa cells, and attempted to figure out a novel regulatory mechanism of post-transcriptional modification on gene expression in BCa progression. More specifically, this study could target FTO signaling, representing a promising strategy for the therapy of BCa.

## Methods

### Study subjects collection of human specimens

A total of 15 cancer tissues and adjacent normal tissues (ANTs) were collected from BCa patients (male (10) vs. female (5)) who received tumor resection in the Qinghai Provincial People’s Hospital (Xining, China) from September 2015 to December 2019. The patients’ mean age was 53.71 ± 16.44 years old. All specimens were immediately stored at −80°C. The inclusion criteria were as follows: 1) patients who were pathologically diagnosed with BCa; 2) patients who did not undergo radiotherapy, chemotherapy or biological therapy preoperatively; 3) patients with complete clinicopathological and follow-up data. All BCa specimens were pathologically confirmed in the Department of Oncology of Qinghai Provincial People’s Hospital, and relevant clinical data were collected through the retrospective review of patients’ medical records.

### Dot plot assay

A dot plot was drawn as previously described [17]. In brief, total RNA was isolated by TRIzol reagent. Then, 1 μg mRNA sample dissolved in 1 ml RNA incubation buffer (50% formamide, 20 mM MOPS (pH 7.0), 2.2 M formaldehyde, 1 mM EDTA, and 5 mM sodium acetate) was denatured at 65°C for 5 min. After that, 200 ng RNAs were added onto an Amersham Hybond-N+ membrane (USEN, Shanghai, China), followed by ultraviolet (UV)-crosslinking for 5 min, and washing with phosphate-buffered saline with Tween-20 (PBST). Afterward, membranes were stained with 0.02% methylene blue (Coweldgen Scientific Co., Ltd., Shanghai, China) to assess the total RNA content. Membranes were blocked with 5% nonfat dry milk, and incubated with the m6A methylation antibody (1:500; Novus Inc., Centennial, CO, USA) at 4°C overnight, and mouse immunoglobulin G (IgG) horseradish peroxidase (HRP)-conjugated antibody for 1 h at room temperature, followed by visualization by an imaging system (Tanon, Shanghai, China).

### Cell culture

Human BCa cell lines (HT-1197 and HT-1376) were purchased from the Cell Bank of Shanghai Institutes of Biological Sciences, Chinese Academy of Sciences (Shanghai, China). A Dulbecco’s modified Eagle’s medium (DMEM; HyClone, Logan, UT, USA) supplemented with 10% fetal bovine serum (FBS; HyClone), 100 U/mL penicillin, and 100 U/mL streptomycin was used to culture cells in an incubator at 37°C in presence of 5% CO2. BCa cells were passaged and sub-cultured into 6-well plates based on cell density adjustment. After that, plasmids or nucleotides were transfected into BCa cells using the Lipofectamine 2000 kit according to the manufacturer’s instructions (Invitrogen, Carlsbad, CA, USA).

## Transfection

Establishment of FTO ectopic plasmid and SFPQ siRNA (GGCACGUUUGAGUACGAAUAU) was undertaken by the Shanghai Sangon Biotechnology Co., Ltd. (Shanghai, China) [18]. Transfections were performed with the Lipofectamine 2000 (Invitrogen) according to the manufacturer’s protocol.

### Cell proliferation assay

Cell proliferation assay was conducted using the cell counting kit-8 (CCK-8) system (USEN). In brief, BCa cells transfected with plasmids or nucleotides were sub-cultured into 96-well plates at a density of 1 × 10^4^ cells/well, and were then incubated for 24 h. Subsequently, optical density (OD) values were evaluated by the absorbance at 490 nm using an Epoch Microplate Reader (BioTek, Winooski, VT, USA). The experiment was repeated three times.

### Transwell migration and invasion assays

The transwell migration and invasion assays were performed using transwell chambers, as described previously [18]. Briefly, HT‐ 1197 and HT‐ 1376 cells were sub-cultured into uncoated and Matrigel-coated upper chambers (BD Biosciences, Franklin Lakes, NJ, USA) for migration and invasion assays, respectively. Moreover, 5 × 10^5^ cells were suspended in the serum-free DMEM and seeded into the upper chamber, and the lower chamber was filled with the complete medium, containing 10% FBS. Cells were stained with 0.5% crystal violet and visualized under an inverted microscope after 24-h incubation. Ten randomly selected fields of vision were observed under a light microscope to count the number of cells.

### Apoptosis assay

The cell apoptosis assay was carried out using Annexin V-APC/PI apoptosis kit (USEN) according to the manufacturer’s instructions. Briefly, the transfected cells were harvested and re-suspended in the binding buffer. Then, 5 μl Annexin V-FITC and 10 μl propidium iodide were added to incubate for 20 min at room temperature in dark conditions. Subsequently, the cells were analyzed by a flow cytometer (BD Biosciences). The experiment was repeated three times.

### Western blot (WB) assay

Total protein was extracted by the RNA immunoprecipitation assay (RIPA) lysis buffer (USEN) supplemented with phenylmethylsulfonyl fluoride (PMSF). The total protein concentration was measured by the bicinchoninic acid (BCA) protein assay kit (USEN), and the sample volume was adjusted to 30 µg protein/lane with deionized water. The samples were subjected to sodium dodecyl sulfate-polyacrylamide gel electrophoresis (SDS-PAGE), and were then transferred onto polyvinylidene fluoride (PVDF) membranes (USEN). The membranes were blocked with 5% skimmed milk for 1 h at room temperature and incubated with FTO (catalog no. 27,226-1-AP; Proteintech, Chicago, IL, USA), CSNK2A2 (catalog no. ab238728; Abcam, Cambridge, UK), ITGA6 (catalog no. ab181551; Abcam), NOTCH1 (catalog no. ab52627, Abcam), and glyceraldehyde 3-phosphate dehydrogenase (GAPDH) (catalog no. 60,004-1-lg; Proteintech) antibodies at 4°C overnight. Afterward, the membranes were probed with the goat anti-rabbit IgG antibody conjugated to HRP (Proteintech) at 37°C for 1 h at room temperature. Subsequently, the bands were visualized using the enhanced chemiluminescence (ECL), and the bands were visualized by a gel imaging system (Tanon). The gray values were calculated using the ImageJ software (National Institutes of Health, Bethesda, MD, USA), and compared with the internal reference to calculate the relative ratio. The experiment was repeated three times.

### RIP assay

The binding affinities of m6A, FTO, and SFPQ on target genes were detected by the RIP kit (Millipore, Burlington, MA, USA). BCa cells were washed with pre-cooled phosphate-buffered saline (PBS). Cold lysis buffer was added and the products were lysed and collected on ice. After removal of the supernatant, the beads were incubated with m6A (catalog no. 56593S; Cell Signaling Technology, Inc., Danvers, MA, USA) and FTO/SFPQ (catalog no. 15,585-1-AP; Proteintech) antibodies for 30 min at room temperature, and rabbit normal IgG (Abcam) was used as the negative control. Subsequently, the cell lysate was further incubated with RIPA lysis buffer, containing magnetic beads coated with an anti-rabbit primary antibody at 4°C for 2 h. RNA was extracted after proteinase K digestion and used for the subsequent quantitative reverse transcription-polymerase chain reaction (RT-qPCR) assay.

### RT-qPCR

Total RNA was extracted from tissues and cells using the TRIzol Reagent (Invitrogen). Then, the extracted RNA was reversely transcribed into the complementary DNA (cDNA) using the PrimeScript™ RT Reagent kit (Takara Biotechnology Inc., Otsu, Shiga, Japan) in accordance with the manufacturer’s instructions. Subsequently, the RT-qPCR was performed using the SYBR® Premix Ex Taq™ II (Tli RNaseH Plus) kit (Takara Biotechnology Inc.) based on the manufacturer’s instructions. The reactions were completed under the following conditions: at 94°C for 2 min, and with 50 cycles of 94°C for 5 s, 58°C for 10 s, 72°C for 30 s, and 72°C for 10 min. The relative expression was calculated using the 2^−ΔΔCT^ method, and GAPDH was used as the internal reference. The experiment was repeated three times. All the primers were synthesized by Coweldgen Scientific Co., Ltd. The following sequences were designed:

FTO:

F: TCCCCAGGGTTGGGATGGGTTCATC,

R: AGGATCCCTGCCTTCGAGATGAGAGT;

MALAT1:

F: ACACTTTAATCTTCCTTCAAAAGGT,

R: AACAACTCGCATCACCGGAATTCGATCACCTTC;

CSNK2A2:

F: TATGTATGAACTACTTAAAGCTCTGG,

R: AATGGTTCCCTTCGAAAGATCATGCTTG;

ITGA6:

F: ATGGGGGTCACCGTCCAGAGCCAAG,

R: CAAAGATGTTCAATGTCAAAAAAAGT;

NOTCH1:

F: CCTGCCTGCACAATGGCCGCTGCCTG,

R: CGGCAGGTGAAGCCATTGATGCCGTCC;

GAPDH:

F: GGAGCGAGATCCCTCCAAAAT,

R: GGCTGTTGTCATACTTCTCATGG.

For RIP-qPCR assay,

MALAT1_5ʹ:

F: CGCAGCCTGCAGCCCGAG,

R: TCAGAGGGGACCTGCCTTCAGG;

MALAT1_3ʹ:

F: TGGTGTCGAGGTCTTTGGTGGGT,

R: GCTTCCGCTAAGATGCTAGCTTGGCCAA;

CSNK2A2_5ʹ:

F: GGGCAGCAGGGCCCGGGTCTACGC,

R: CTATATTTTCCCCGACCAAGTTTTCGA;

CSNK2A2_3ʹ:

F: AAGGAGCAGTCCCAGCCTTGTGCA,

R: CGTTAAGACGTTTGATTTGGTTCTT;

ITGA6_5ʹ:

F: GCTCCTGTCCCGGCTCGGCGCAGCC,

R: GTACAGCCCTCCCGTTCTGTTGGCTC;

ITGA6_3ʹ:

F: GCTCCTGTCCCGGCTCGGCGCAGCC,

R: GTGCAAAACAGGAGCCTTAGGCTACG;

NOTCH1_5ʹ:

F: CACGGAGGCCTGCGTCTG,

R: CCGTCAGCGTGAGCAGGTC;

NOTCH1_3ʹ:

F: ACAGTAGCCTTGCTGCCAGCGCC,

R: GTGGTGGTGGTGGTGGCTGCAGGCT.

### A nude mouse xenograft model

BALB/cnu/nu mice (4–5 weeks old) were used for the xenograft experiment. The mice were randomly divided into 2 groups (n = 6 for each group) and injected with 5 × 10^6^ HT-1197 cells in control group or FTO plasmid group, respectively. Tumor volume was recorded every 2 days, and it was calculated according to the following formula: length × width [2]/2. The study protocol was approved by the Ethics Committee of the Qinghai Provincial People’s Hospital, and animal experiments were conformed to the Guide for the Care and Use of Laboratory Animals (National Institutes of Health (NIH), Bethesda, MD, USA; NIH Publication No. 8023, revised in 1978).

### Immunohistochemistry (IHC)

IHC was performed on paraffin-embedded mouse tissue sections (5 mm) to determine Ki-67 expression. The slides were incubated with anti–Ki-67 antibody (1:500, catalog no. ab15580, Abcam) overnight at 4°C. HRP Detection System (ZSGB-bio, Beijing, China) and 3,3ʹ-Diaminobenzidine (DAB) Substrate kit (ZSGB-bio) were subsequently used. Then, the sections were dehydrated and mounted, and observed under a light microscope (Olympus, Tokyo, Japan). The percentage of Ki-67–positive was measured using Image-Pro Plus 6.0 software (Media Cybernetics, Inc., Bethesda, MD, USA).

### Terminal deoxynucleotidyl transferase dUTP nick end labeling (TUNEL) assay

TUNEL assay was used to identify the rate of apoptosis in paraffin-embedded mouse tissue sections (5-mm) with an *in situ* cell death detection kit (Roche, Basel, Switzerland) according to the manufacturer’s instructions. The apoptotic cells were observed under a light microscope (Olympus). The experiment was repeated three times. The rate of apoptosis was measured using IPP 6.0 software.

### Statistical analysis

Experimental raw data were processed using the SPSS 22.0 software (IBM Corp., Armonk, NY, USA). The data were displayed as mean ± standard deviation (SD). The paired t-test was used for comparing the gene expression between cancer tissues and ANTs, and the one-sample t-test was employed for comparing the gene expression between the other two groups. One-way analysis of variance (ANOVA) was utilized for comparing the gene expression among multiple groups, and the pairwise comparisons were made by the post-hoc test. The Pearson’s correlation analysis was performed to find out the correlation between m6A expression and FTO mRNA expression in BCa patients. The Kaplan-Meier method and the log-rank test were utilized to compare the overall survival (OS) rate between the FTO high-expression group and the FTO low-expression group. P < 0.05 was considered statistically significant.

## Results

### Detection of the FTO expression in BCa tissues and ANTs in vivo

**Figure 1.**
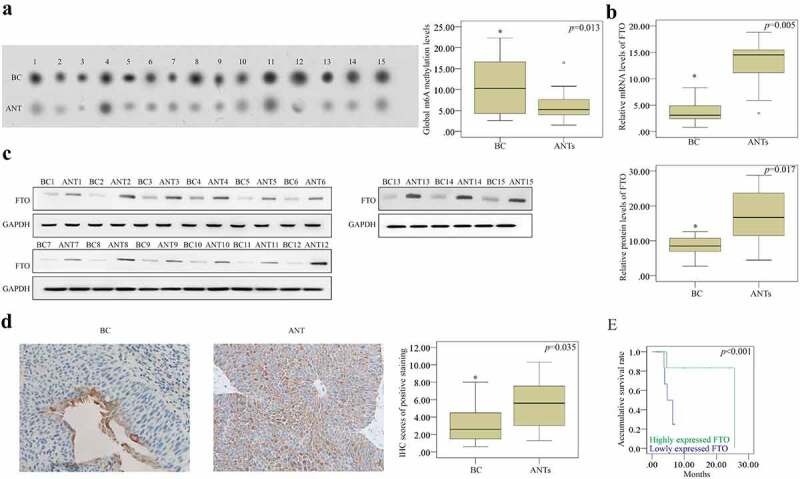
The relationship between the FTO expression and the m6A RNA methylation. (a). the m6A RNA methylation level was detected by dot plot assay and it was elevated in BCa tissues compared to that in the ANTs. (b-c). the FTO expression was up-regulated in BCa group according to the results of RT-qPCR and western blotting (d). determination of the FTO expression in BCa tissues and ANTs by IHC (scale bar, 100 µm; magnification, 200×), showing that the FTO expression increased in BCa tissues compared with that in ANTs. (e) the prognostic effect of FTO expression on overall survival rate of BCa patients determined using the kaplan-meier method, indicating that FTO could be advantageous to prolong overall survival of BCa patients. BCa: bladder cancer; ANTs: adjacent normal tissues. all the data are presented as mean ± standard error of the mean. ‘*’ represents *P* < 0.05

In the current study 15 human BCa tissues and ANTs were utilized to detect the m6A level using WB assay. We noticed that the level of m6A RNA methylation was substantially increased in the BCa tissues compared with that in the ANTs from randomly selected specimens (Figure 1(a)). Moreover, RT-qPCR (Figure 1(b)), WB (Figure 1(c)), and IHC (Figure 1(d)) assays were performed to identify the FTO expression. Compared with the ANTs, the FTO expression was down- regulated in BCa tissues. Owing to the limited number of samples, samples were only classified into low malignancy (stages I and II) and high malignancy (stages III and IV) subtypes based on pathological grades, and the Chi-square test indicated that the low expression of FTO, as detected by IHC, was associated with the high incidence of BCa (χ2 = 6.418, P = 0.017). Moreover, the Kaplan-Meier analysis exhibited that the expression of FTO was positively correlated with the OS rate of BCa patients (Figure 1(e)). Besides, the FTO expression was found as an independent prognostic factor for tumor grading (hazard ratio (HR) = 8.214, 95% confidence interval (CI): 1.517–5.028, P = 0.009) and OS rate (HR = 4.525, 95% CI: 2.285–6.133, P = 0.002) in BCa patients. Collectively, the reduced FTO expression increased the m6A level, indicating a poor prognosis of BCa patients.

### Anti-carcinogenic effects of FTO expression on BCa cells

**Figure 2.**
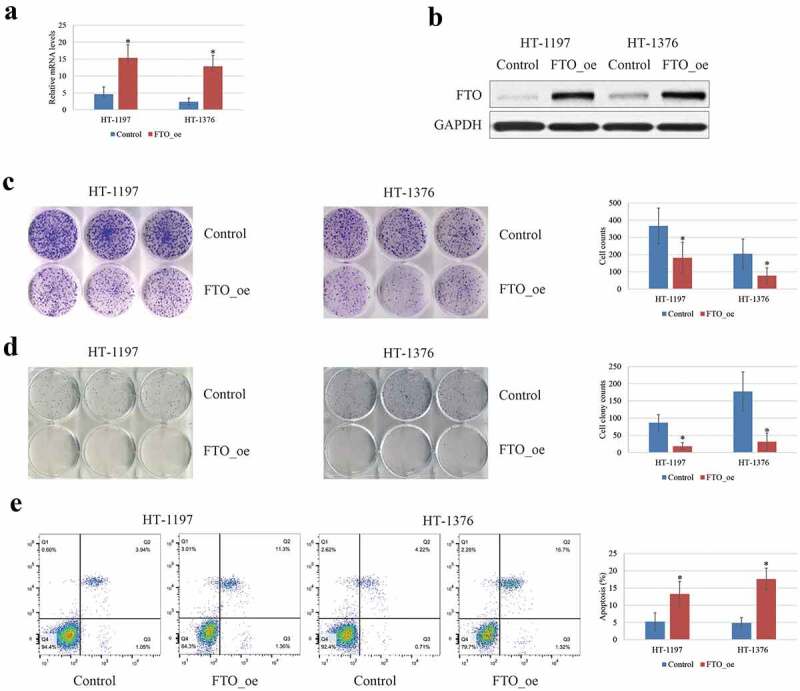
The tumor-suppressive effects of FTO on BCa cells. (a-b). The FTO expression was up-regulated following transfection with FTO plasmid in BCa cell lines by RT-qPCR and western blotting. (c). MTT analysis showed that overexpression of FTO could inhibit cell viability of BCa cells. (d). Transwell invasion analysis showed that the capability of invasion was reduced after transfection with FTO plasmid. (e). overexpression of FTO could enhance the apoptosis of BCa cells by flow cytometry. all the data are presented as mean ± standard error of the mean. ‘*’ represents *P* < 0.05

The effects of the FTO expression on tumor progression and malignancy were studied in HT‐1197 and HT‐1376 cells *in vitro*. It was revealed that overexpression of FTO in the HT‐1197 and HT‐ 1376 cells (Figure 2(a-b)) significantly decreased the cell proliferation (Figure 2(c)) and invasion (Figure 2(d)) abilities compared with the negative controls, whereas the cell apoptosis was markedly enhanced (Figure 2(e)). Taken together, the FTO exhibited a tumor-suppressive effect on BCa cells.

### Knockdown of SFPQ alleviated the effects of FTO overexpression on m6A RNA methylatio in BC cells

**Figure 3.**
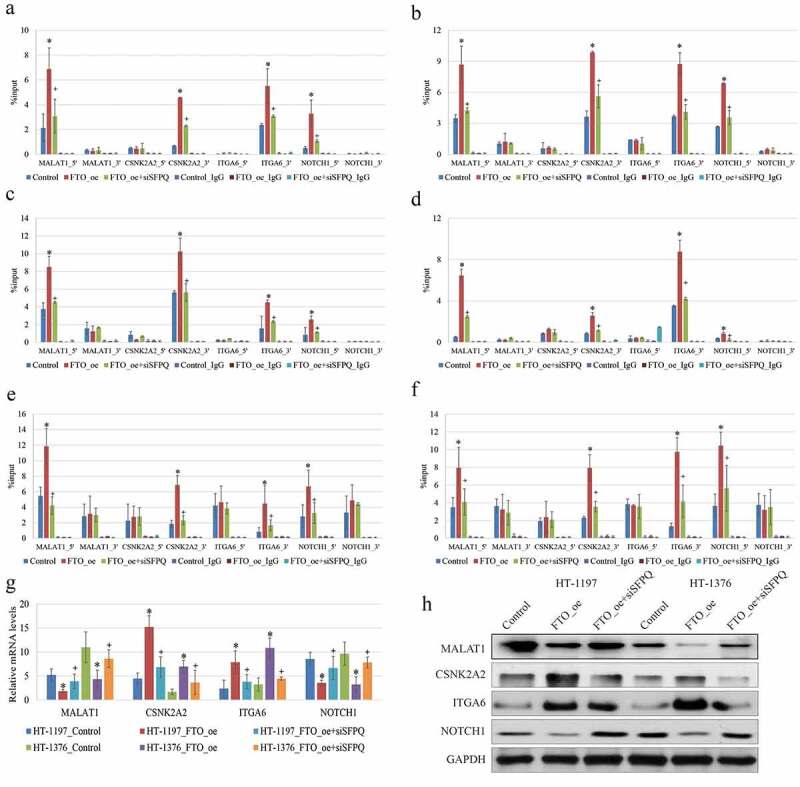
Knockdown of SFPQ alleviated the effects of FTO overexpression on m6A RNA methylatio in BC cells. (a-b). RIP-qPCR analysis showed that the proportion of SFPQ was substantially different between 5/3ʹUTRs of MALAT1, NOTCH1, CSNK2A2, and ITGA6genes. RIP-qPCR showed that the enrichment of SFPQ was extremely low in sites where the remarkably dynamic changes of m6A methylation appeared after FTO transfection. (c-d). FTO_oe: FTO over-expression; siSFPQ: SFPQ knockdown. All the data are presented as mean ± standard error of the mean. ‘*’ represents *P* < 0.05 compared to control group, and ‘+’ denotes *P* < 0.05 compared to FTO_oe group. (e-f). The changes of m6A at 5ʹ UTR of MALAT1 and NOTCH1 transcripts, and at 3ʹ UTR of CSNK2A2 and ITGA6 transcripts in HT-1197 (left) and HT-1376 (right) cells with overexpression of FTO and knockdown of SFPQ, indicating that enrichments of m6A-specific antibody were all reduced on the transcripts of these genes in BCa cells with overexpression of FTO compared to negative controls. (g-h). The MALAT1, NOCH1 mRNA and protein levels in BCa cells were down-regulated after transfection with FTO plasmid, while they were up-regulated after combination with SFPQ siRNA. The CSNK2A2, ITGA6 mRNA and protein in BCa cells were up-regulated after transfection with FTO plasmid, while they were down-regulated after combination with SFPQ siRNA

A number of scholars identified one RNA-binding protein, SFPQ, directly interacting with FTO, and explored the regulatory mechanism that could assist FTO to gain a substrate specificity [19]. To figure out the mechanism underlying the tumor-suppressive effect of FTO expression on BCa cells, we detected the expressions of the known differentially m6A methylated genes, including MALAT1 [12], CSNK2A2 [20], ITGA6 [21], and NOTCH1 [22], which were found to correlate with BCa progression in previous studies. In the present research, RT-qPCR data showed that the occupancy of SFPQ was substantially different from 5/3ʹUTRs of the four target genes (Figure 3(a-b)). The enrichment of SFPQ was extremely low in sites where the remarkably dynamic changes of the m6A RNA methylation appeared after FTO transfection. We also observed that the enrichment of SFPQ was noticeably coincident with the FTO expression in HT-1197 and HT-1376 cells (Figure 3(c-d)). Furthermore, the knockdown of SFPQ attenuated the binding affinity of FTO, substantially alleviated the effects of FTO overexpression on m6A RNA methylation, and downregulated the expressions of the four genes in BCa cells. With the aid of data extracted from the SRAMP server (http://www.cuilab.cn/sramp), the RT-qPCR showed the decreased enrichment of m6A-containing RNA using m6A RNA methylation antibody on the transcripts of these genes in HT‐1197 and HT‐1376 cells with the overexpression of FTO compared with the negative controls (Figure 3(e-f)). In addition, we noticed that the changes of m6A methylation mainly appeared at 5ʹ UTR of MALAT1 and NOTCH1 transcripts, and at 3ʹ UTR of CSNK2A2 and ITGA6 transcripts responding to the overexpression of FTO. However, the expressions of these genes were not consistent. The expressions of MALAT1 and NOTCH1 were reduced, while those of CSNK2A2 and ITGA6 were elevated when FTO was overexpressed (Figure 3(g-h)). Taken together, SFPQ could influence the FTO-mediated m6A RNA methylation, eventually affecting the gene expression.

### Overexpression of FTO could inhibit the growth of BCa cells in vivo

**Figure 4.**
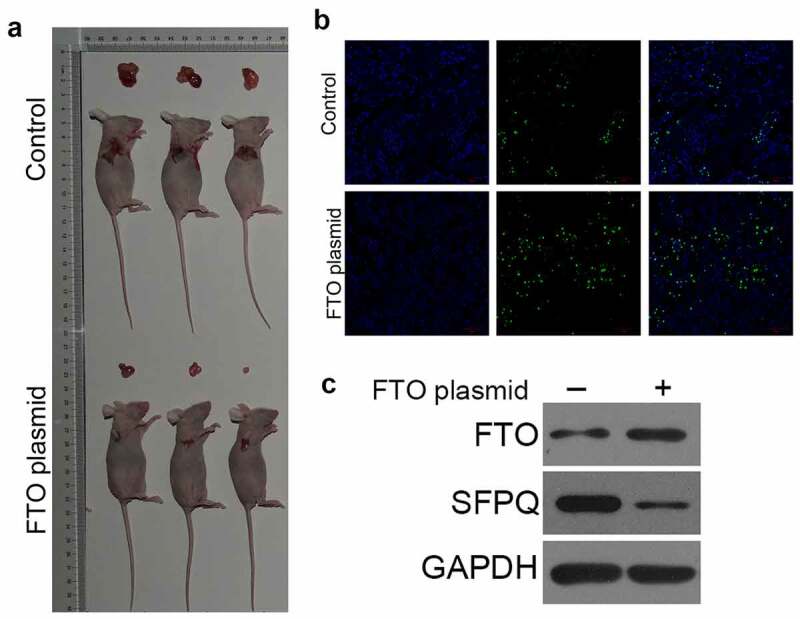
Over-expression of FTO could inhibit the growth of BC cells in vivo A. tumor xenograft mouse model showed that the tumor volume was significantly reduced in mice treated with FTO plasmid. B. tunel was used to determine cell apoptosis, showed that FTO plasmid enhanced cell apoptosis. C. western blot analyzed the expression of FTO and SFPQ

To explore the effects of FTO on the growth of BCa cells *in vivo*, we established a tumor xenograft mouse model. As shown in Figure 4, the tumor volume was significantly reduced in mice treated with FTO plasmid. In addition, the tumor inhibitory rate was higher in mice in the FTO plasmid group (Figure 4(a)). In addition, in the FTO plasmid group, the cell proliferation decreased and the cell apoptosis increased compared to the control group (Figure 4(b)). Furthermore, treatment with FTO plasmid significantly up-regulated FTO expression and down-regulated SFPQ expression (Figure 4(c)). These results confirmed our findings from the *in vitro* studies, approving the function of FTO *in vivo*.

## Discussion

As for BCa, one of the leading causes of cancer-related deaths in adults, it has been reported that approximately 400,000 new BCa cases are annually diagnosed worldwide. Surgery, chemotherapy, and radiation therapy are the main therapeutic approaches for BCa, while its recurrence rate is still noticeable [23]. The occurrence and development of BCa could be influenced by a large number of complicated internal and external factors. Recently, a number of scholars identified novel biomarkers to predict disease recurrence to better understand the molecular mechanism of BCa [24].

New evidence shows that these m6a regulatory protein changes promote the proliferation and survival of cancer cells, the initiation and progression of tumors by inducing the expression of key oncogenes [25]. As a key demethylase, FTO regulates the stability of cellular mRNA by removing mA residues in mRNA, and it has been shown to be related to the risk of cancer [26]. Functional studies on m6A RNA methylation have characterized the RNA demethylase FTO, and announced that m6A RNA methylation process is a continuously dynamic and reversible process. A recent study failed to suggest an association between FTO polymorphism and the increased risk of cancer, even though FTO polymorphism was marginally associated with the occurrence of pancreatic cancer [27]. FTO is highly expressed in many types of cancer such as human cervical squamous cell carcinoma tissue and gastric cancer [28,29]. Nevertheless, abnormal changes of FTO expression in BCa tissues have resulted in controversial outcomes [12,30]. Although the differences in the collection of tissue samples and patients’ clinical conditions may partially explain the discrepancy, it is noteworthy to consider the expressions of a distinct set of target genes regulated by the FTO expression. Moreover, the deficiency of another m6A demethylase (ALKBH5) and the m6A methyltransferases (METTL3 and METTL14) may affect the level of m6A in BCa patients [20, 21].

From the results of the present research, we observed that the presence of the m6A RNA methylation mediated by FTO in different UTRs of the four target genes could lead to different gene expressions. It is noteworthy that SFPQ could influence the FTO-mediated m6A RNA methylation, eventually affecting the gene expression. SFPQ, as an RNA-binding protein (RBP), is ubiquitously and abundantly expressed, and plays important roles in DNA damage repair and nuclear paraspeckle formation [31]. In addition, the dysfunction of SFPQ is associated with pathological features evidenced by multiple disease-associated RBPs, including alternative RNA splicing or cytoplasmic localization. Nevertheless, the roles of SFPQ in the detection of different m6A sites, influencing the occurrence of BCa and other diseases, should be assessed in the future researches.

## Conclusions

In summary, FTO was identified as an inhibitory gene involved in the tumorigenesis of BCa. The low expression of FTO was detected in BCa tissues, suggesting that FTO is a significant hallmark for either the clinical diagnosis or prognosis of BCa. Furthermore, FTO could suppress the tumorigenesis of BCa, specifically the cell proliferation and invasion abilities *in vitro*. Additionally, the knockdown of SFPQ attenuated the binding affinity of FTO, substantially alleviated the effects of the FTO overexpression on m6A RNA methylation, and downregulated the expressions of the four genes in BCa cells. This study may benefit to highlight the role of FTO as a diagnostic or prognostic biomarker, as well as being a therapeutic target for BCa.

## Data Availability

The data that support the findings of this study are available from the corresponding author on a reasonable request.
